# Identification and functional analysis of the L-ascorbate-specific enzyme II complex of the phosphotransferase system in *Streptococcus mutans*

**DOI:** 10.1186/s12866-016-0668-9

**Published:** 2016-03-22

**Authors:** Xinyu Wu, Jin Hou, Xiaodan Chen, Xuan Chen, Wanghong Zhao

**Affiliations:** Department of Stomatology, Nanfang Hospital and College of Stomatology, Southern Medical University, Guangzhou, Guangdong China; Department of Stomatology, the Second Affiliated Hospital of Shantou University, Shantou, Guangdong China

**Keywords:** *Streptococcus mutans*, Phosphotransferase system, L-ascorbate, Aciduricity, Acidogenesis, Biofilm formation, Extracellular polysaccharides

## Abstract

**Background:**

*Streptococcus mutans* is the primary etiological agent of human dental caries. It can metabolize a wide variety of carbohydrates and produce large amounts of organic acids that cause enamel demineralization. Phosphoenolpyruvate-dependent sugar phosphotransferase system (PTS) plays an important role in carbohydrates uptake of *S. mutans*. The *ptxA* and *ptxB* genes in *S. mutans* encode putative enzyme IIA and enzyme IIB of the L-ascorbate-specific PTS. The aim of this study was to analyze the function of these proteins and understand the transcriptional regulatory mechanism.

**Results:**

*ptxA*^−^, *ptxB*^−^, as well as *ptxA*^−^*, ptxB*^−^ double-deletion mutants all had more extended lag phase and lower growth yield than wild-type strain UA159 when grown in the medium using L-ascorbate as the sole carbon source. Acid production and acid killing assays showed that the absence of the *ptxA* and *ptxB* genes resulted in a reduction in the capacity for acidogenesis, and all three mutant strains did not survive an acid shock. According to biofilm and extracellular polysaccharides (EPS) formation analysis, all the mutant strains formed much less prolific biofilms with small amounts of EPS than wild-type UA159 when using L-ascorbate as the sole carbon source. Moreover, PCR analysis and quantitative real-time PCR revealed that *sgaT*, *ptxA*, *ptxB, SMU.273*, *SMU.274* and *SMU.275* appear to be parts of the same operon. The transcription levels of these genes were all elevated in the presence of L-ascorbate, and the expression of *ptxA* gene decreased significantly once *ptxB* gene was knockout.

**Conclusions:**

The *ptxA* and *ptxB* genes are involved in the growth, aciduricity, acidogenesis, and formation of biofilms and EPS of *S. mutans* when L-ascorbate is the sole carbon source. In addition, the expression of *ptxA* is regulated by *ptxB. ptxA*, *ptxB,* and the upstream gene *sgaT*, the downstream genes *SMU.273*, *SMU.274* and *SMU.275* appear to be parts of the same operon, and L-ascorbate is a potential inducer of the operon.

**Electronic supplementary material:**

The online version of this article (doi:10.1186/s12866-016-0668-9) contains supplementary material, which is available to authorized users.

## Background

*Streptococcus mutans* is the primary etiological agent of human dental caries. It can metabolize a wide variety of carbohydrates that exist in the human oral cavity and produce large amounts of organic acids via the glycolytic pathway [1]. These metabolic byproducts cause a substantial drop in the pH of the oral cavity that in turn can result in the demineralization of enamel. The mechanisms of transport and metabolism of carbohydrates by *S. mutans* are therefore crucial to the onset and development of dental caries.

Although there are examples of carbohydrates that are internalized through ATP-binding cassette transporters (ABC transporters) [2], or other pathways [3–5], the dominant, high-affinity, high-capacity mechanism to transport and concomitantly phosphorylate carbohydrates in *S. mutans* is the phosphoenolpyruvate (PEP)-dependent sugar phosphotransferase system (PTS) [6]. More than 14 unique PTS permeases that transport a spectrum of carbohydrates including glucose [7, 8], sucrose [9], mannose [10], sorbitol [11], fructose [12], lactose [13], galactose [14, 15], maltose [16] and nigerose [17] are present in the reference strain *S. mutans* UA159. The PTS is usually composed of two general energy-coupling proteins that participate in the phosphorylation of all PTS substrates—the enzyme I (EI) and histidine-containing phosphocarrier protein (HPr) and a series of substrate-specific permeases, known as enzyme II (EII) complexes, which are directly responsible for the transportation and phosphorylation of the substrates [6]. In most cases, the EII complexes are comprised of three functional domains, A, B, and C, but sometimes a fourth domain, D, is required. The EIIA and EIIB domains are located in the cytoplasm and take part in the phosphorylation of the cognate substrates, while the EIIC and EIID domains act as the transmembrane channel and the sugar-binding site [18]. The PTS phosphorylates carbohydrates at the expense of PEP. During the transport process, the phosphoryl group on PEP is transferred to EI, then to a histidine residue on HPr, then to EIIA and EIIB, and finally to EIIC, forming a sugar-phosphate [18]. To date, a number of sugar-specific PTS of *S. mutans* have been further studied, such as glucose, sucrose, fructose, mannose, sorbitol, etc. Howerer, the study of L-ascorbate-specific PTS of *S. mutans* is little.

Previous studies have reported that some enteric bacteria can ferment and oxidize L-ascorbate under anaerobic conditions [19–21]. The metabolism of L-ascorbate has been described in detail in *Escherichia coli* [22–24], *Lactobacillus* [25] and *Pneumobacillus* [26, 27], but no study has formally shown that *S. mutans* can ferment this compound. In natural environment, the energy supply for growth and survival is often a limiting factor, organisms regularly encounter such energy-limited conditions, and they are forced to scavenge energy from all potential sources. L-ascorbate is abundant in many fruits and vegetables, so to study on the L-ascorbate-specific PTS that oral streptococci can use to obtain carbon sources is important.

Currently, six genes, *ptxA, ptxB, sgaT*, *SMU*.273, *SMU*.274 and *SMU*.275, analogous to the *ula* regulon used by *E. coli* to catabolize L-ascorbate under anaerobic conditions, have been identified in the *S. mutans* genome. These genes encode putative EIIA, EIIB, and EIIC of the L-ascorbate-specific PTS of *S. mutans* and three catabolic enzymes in the pentose phosphate pathway. Recently, the crystal structures of the PtxA (PDB: 3BJV) and PtxB (PDB: 3CZC) proteins have been analyzed [28]. Specific hydrophobic structures between these two proteins allow for efficient transfer of the phosphoryl group from PtxA to PtxB and then to the substrate.

In the present study, we knocked out the putative L-ascorbate-specific EIIA gene (*ptxA*) and EIIB gene (*ptxB*), individually and together, in *S. mutans* UA159, to explore their function. The results indicate that *ptxA* and *ptxB* are involved in growth, aciduricity, acidogenesis, and formation of biofilms and extracellular polysaccharides (EPS) when *S. mutans* is grown with L-ascorbate as the sole carbon source. Moreover, the expression of *ptxA* is regulated by *ptxB. ptxA*, *ptxB,* and the adjacent genes *sgaT, SMU*.273, *SMU*.274 and *SMU*.275 are parts of the same operon, and L-ascorbate is a potential inducer of the operon.

## Methods

### Bacterial strains, plasmids, and culture conditions

The *S. mutans* strains and plasmids used in this study are listed in Table 1. *S. mutans* UA159 and its derivatives were routinely grown in brain-heart infusion (BHI) medium (Hopebio, Qingdao, Shandong, China) or tryptone-vitamin (TV) base medium [29] supplemented with 15 mM L-ascorbate (Sigma, St Louis, MO, USA) or glucose (Sigma, St Louis, MO, USA) as the sole carbon source, which were referred to as TVL medium and TVG medium, respectively. When needed, 1 mg mL^−1^ spectinomycin (Sigma, St Louis, MO, USA) was added to the medium. All bacterial cultures were incubated without agitation in an anaerobic atmosphere (10 % CO_2_, 10 % H_2_, 80 % N_2_) at 37 °C, unless specified otherwise.

**Table 1:** Bacterial strains and plasmids used in this study

Strains or plasmids	Relevant characteristics	Source or reference
Strains		
*S. mutans* UA159	Wild-type, serotype c	Ajdić *et al*., (2002) [4]
*S. mutans ptxA* ^*−*^	UA159 derivative, △*ptxA* ^*−*^, Spe^r^	This study
*S. mutans ptxB* ^*−*^	UA159 derivative, △*ptxB* ^*−*^, Spe^r^	This study
*S. mutans ptxAB* ^*−*^	UA159 derivative, △*ptxA* ^*−*^ and △*ptxB* ^*−*^, Spe^r^	This study
*S. mutans CptxA* ^*−*^	*S. mutans ptxA* ^*−*^ carrying pDL278:*ptxA*, Spe^r^	This study
*S. mutans CptxB* ^*−*^	*S. mutans ptxB* ^*−*^ carrying pDL278:*ptxB*, Spe^r^	This study
*S. mutans CptxAB* ^*−*^	*S. mutans ptxAB* ^*−*^ carrying pDL278:*ptxAB*, Spe^r^	This study
Plasmids		
pFW5	Commercial cloning vector, Spe^r^	Podbielski A *et al*., (1996) [52]
pDL278	Shuttle vector, Spe^r^	LeBlanc & Lee (1991) [53]
pDL278:*ptxA*	Shuttle vector carrying *ptxA*, Spe^r^	This study
pDL278:*ptxB*	Shuttle vector carrying *ptxB*, Spe^r^	This study
pDL278:*ptxAB*	Shuttle vector carrying *ptxA* and *ptxB*, Spe^r^	This study

*Spe* ^*r*^ spectinomycin resistance

### Construction of *ptxA*^−^, *ptxB*^−^, and *ptxA*^−^, *ptxB*^−^ double deletion mutants and complemented strains

The procedure for generating the plasmid for construction of a *ptxA*^*−*^ strain was described previously [30]. Briefly, the 5’ and 3’ regions flanking the *ptxA* gene were amplified from the genomic DNA of *S. mutans* UA159 by polymerase chain reaction (PCR) using the primers shown in Additional file 1: Table S1. Following proper restriction enzyme digestions, the flanking regions were cloned into two multiple cloning sites of plasmid pFW5 to generate pFW5A. Subsequently, plasmid pFW5A was used to transform the wild-type strain UA159, which resulted in replacement of the *ptxA* gene by a non-polar spectinomycin resistance (Spe^r^) marker via allelic exchange. The transformation was carried out in BHI medium in the presence of 10 % heat-inactivated horse serum and 100 nM competence-stimulating peptide (CSP) [31]. Spectinomycin-resistant transformants were isolated, further confirmed by PCR and sequencing, and named *S. mutans ptxA*^*−*^ strain. A similar technique was used to construct a *ptxB*^−^ deletion mutant and a *ptxA*^−^, *ptxB*^−^ double deletion mutant, named *S. mutans ptxB*^*−*^ strain and *S. mutans ptxAB*^*−*^ strain, respectively. For complementation of mutants, the *ptxA, ptxB,* and *ptxA*–*ptxB* coding sequences, plus the P_*gtfB*_ promoter [32], were amplified by PCR, digested and cloned directly into shuttle vector pDL278 to generate pDL278:*ptxA*, pDL278:*ptxB*, pDL278:*ptxAB*, respectively. After sequence confirmation, the correct plasmids were used for transformation of *S. mutans ptxA*^*−*^, *ptxB*^*−*^, and *ptxAB*^*−*^ strains, generating complemented strains *S. mutans CptxA*^*−*^*, CptxB*^*−*^ and *CptxAB*^*−*^, respectively.

### Bacterial growth rates

To measure the growth rates of *S. mutans* when using L-ascorbate or glucose as the sole carbon source, wild-type strain UA159 was grown in BHI medium overnight, and the optical density at 600 nm (OD_600_) of the cultures was adjusted to 1.0. The adjusted cultures were inoculated 1:100 into fresh TVL or TVG medium. Data for plotting growth curves were collected by measuring changes in OD_600_ at 2 h intervals using a spectrophotometer over a total period of 48 h. To compare the growth rates among UA159 and its derivatives, overnight cultures were diluted 1:100 into fresh TVL medium, and OD_600_ values were measured at 2 h intervals for a total of 72 h.

### Acid production assay

Overnight cultures of wild-type *S. mutans* UA159 and its derivatives in BHI medium were diluted 1:100 with fresh TVL medium or fresh BHI medium and then incubated at 37 °C in an anaerobic atmosphere for 24 h and 48 h. The pH of the supernatant in the media was measured at the beginning, and after 24 h or 48 h of incubation. The acidogenesis ability was calculated as the difference in pH values measured at specific incubation times (ΔpH).

### Acid killing assay

The ability of the mutants to tolerate acid stress was determined by acid killing assays, as described previously [33, 34]. Briefly, *S. mutans* strains were grown in TVL medium until OD_600_ ≈ 0.3, harvested by centrifugation at 3800 × g at 4 °C for 10 min, washed once with 0.1 M glycine (Sigma, St Louis, MO, USA), pH 7.0, then the cell pellets were resuspended in fresh TVL medium that was adjusted to pH 5.0 with HCl to undergo an adaptive acid tolerance response. Following an additional hour of incubation, cells were harvested, washed and subjected to acid killing by incubating the strains in 0.1 M glycine, pH 2.8, for 0, 15, 30, and 45 min. The surviving cells were appropriately diluted, plated on BHI agar, and incubated in an anaerobic atmosphere at 37 °C for 48 h.

### Biofilm and EPS formation analysis

To evaluate the biomass and structure of the biofilms with confocal laser scanning fluorescence microscopy, *S. mutans* UA159 and its derivatives were incubated in BHI medium overnight, and new cultures were inoculated by diluting them 1:100 into fresh TVL medium and dispensing 5 mL aliquots into 6-well plates (Corning, NY, USA) with coverslips in each well. After 120 h of 37 °C anaerobic incubation, the formed biofilms were washed gently twice with sterile PBS to remove unbound bacteria and stained with SYTO9 (Molecular Probes, Eugene, OR, USA) for 15 min at room temperature in a dark room. After SYTO9 removal, biofilms were incubated in calcofluor white (Sigma, St Louis, MO, USA) to stain the EPS under identical conditions. Then the biofilms were washed gently twice with sterile PBS again and examined with an Olympus Fluoview FV10i confocal microscope (Olympus, Tokyo, Japan). For the detection of SYTO9 (green), we used the 488 nm line of the argon laser. For calcofluor white (blue), we used the 351 nm line. At least five independent fields were collected at 100× magnification per experiment and three independent experiments were performed. Image J was used to calculate the area that the biofilms covered.

### PCR analysis and quantitative real-time PCR

To characterize the mechanism regulating expression of the *ptxA* and *ptxB* genes, total RNA was extracted and purified. Briefly, an overnight culture of *S. mutans* UA159 was added to TVL medium or TVG medium and grown to late exponential phase. The cells were disrupted with liquid nitrogen and the RNA was extracted with RNAiso reagent (Takara, Otsu, Shiga, Japan) and treated with DNase I (Thermo Scientific, Utena, Lithuania). After confirming the absence of DNA by PCR, the conversion of RNA into cDNA was carried out using the PrimeScript RT Master Mix protocol (Takara, Otsu, Shiga, Japan). PCR was performed on cDNA templates with specific primers that span the sequences *SMU*.268 to *sgaT*, *sgaT* to *ptxB*, *ptxB* to *ptxA*, *ptxA* to *SMU*.273, *SMU*.273 to *SMU*.274, *SMU*.274 to *SMU*.275 and *SMU*.275 to *SMU*.277 (Additional file 1: Table S1), using DNA of *S. mutans* UA159 as a positive control [35, 36]. To evaluate the expression of *ptxA*, *ptxB* and their adjacent genes under the influence of 15 mM L-ascorbate (with 15 mM glucose used as control), quantitative real-time PCR (qRT-PCR) was performed with specific primers (Additional file 1: Table S1) using the SYBR Premix Ex Taq Kit protocol (Takara, Otsu, Shiga, Japan). The qRT-PCR amplification with primers to the 16S rRNA gene was used as a reference for normalization. Non-template controls were included to confirm the absence of primer-dimer formation. In addition, expression of *ptxA* gene in wild-type UA159 and *ptxB*^*−*^ strain was also evaluated by qRT-PCR.

### Statistical analysis

Quantitative data were analyzed using the Independent-samples *t*-test or One-way ANOVA test, and a *P* value < 0.05 indicated statistically significant differences.

## Results

### Deletion of *ptxA* or *ptxB* causes major defects in bacterial growth rates

**Fig. 1:**
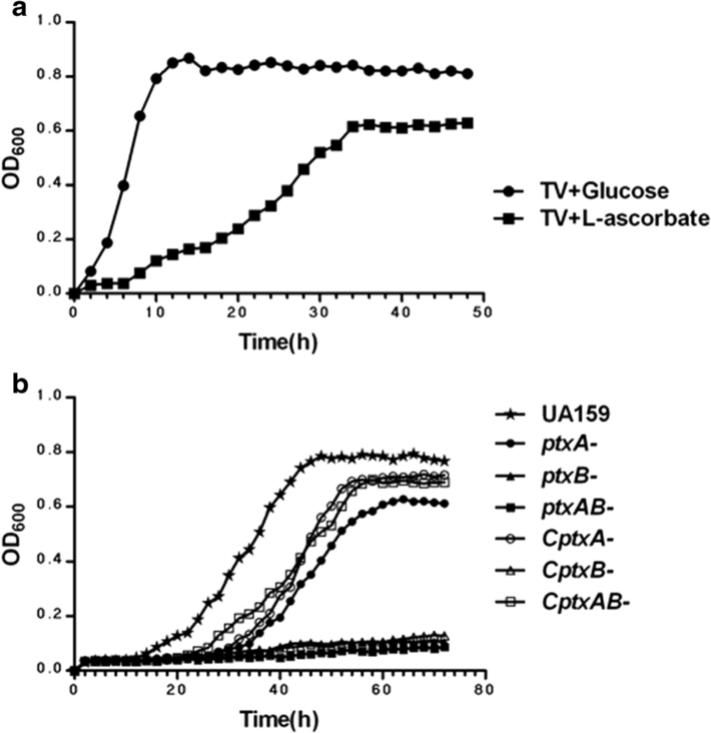
Bacterial growth rates. **a** Growth of *S. mutans* UA159 incubated in TV medium supplemented with 15 mM glucose (●) or L-ascorbate (■). **b** Growth of wild-type UA159 (★), *ptxA* ^*−*^ strain (●), *ptxB* ^*−*^ strain (▲), *ptxAB* ^*−*^ strain (■), *CptxA* ^*−*^ strain (○), *CptxB* ^*−*^ strain (△) and *CptxAB* ^*−*^ strain (□) incubated in TV medium supplemented with 15 mM L-ascorbate. Samples were all grown at 37 °C for more than 48 h under anaerobic conditions and monitored every 2 h at 600 nm (OD_600_). The data presented here are the average of three independent experiments performed in triplicate

In initial experiments, we tested the growth of the wild-type *S. mutans* UA159 under anaerobic conditions in TV base medium supplemented with various concentrations of L-ascorbate as the sole carbon source. We found that, to some extent, the growth yield increased with increase in the L-ascorbate concentration. However, higher concentrations retarded or even stopped growth. Consequently, considering the terminal yield of bacteria and the extent of the lag phase, we selected 15 mM as the optimal concentration of L-ascorbate. When grown in the two different media, *S. mutans* reached a growth plateau in TVL medium at about 36 h, and in TVG medium at 14 h. The maximal culture density (OD_600_) in TVL medium was found to be reduced by more than one third of that in TVG medium (Fig. 1a). These results indicated that L-ascorbate could act as a carbon source for *S. mutans* to survive under anaerobic conditions, but not as effectively as glucose. The slow induction in TVL may in part account for its longer lag period and lower terminal yield.

Deletions of the *ptxA* or *ptxB* genes impaired the ability of *S. mutans* to grow when using L-ascorbate as the sole carbohydrate (Fig. 1b). After 72 h of 37 °C anaerobic incubation, wild-type UA159 reached an OD_600_ of 0.8 and its lag phase was 12 h. However, compared with wild-type UA159, the *ptxA*^*−*^ strain had an extended lag phase and decreased growth yield. The lag phase of *ptxA*^*−*^ strain was 22 h and its maximal culture density was only 0.6 approximately. In addition, the growth of *ptxB*^*−*^ and *ptxAB*^*−*^ strains was decreased even more substantially. In the 72 h of incubation, they could hardly grow. As expected, the presence of the recombinant plasmids pDL278:*ptxA* and pDL278:*ptxAB* restored the anaerobic growth of the *CptxA*^*−*^ and *CptxAB*^*−*^ strains on L-ascorbate, although the growth rates and the OD_600_ were lower after 72 h of incubation when compared to those of the wild-type UA159. However, the *CptxB*^*−*^ strain could not be complemented by inclusion of the plasmid pDL278:*ptxB*.

### *ptxA* and *ptxB* deletions resulted in reduced acidogenesis

**Fig. 2:**
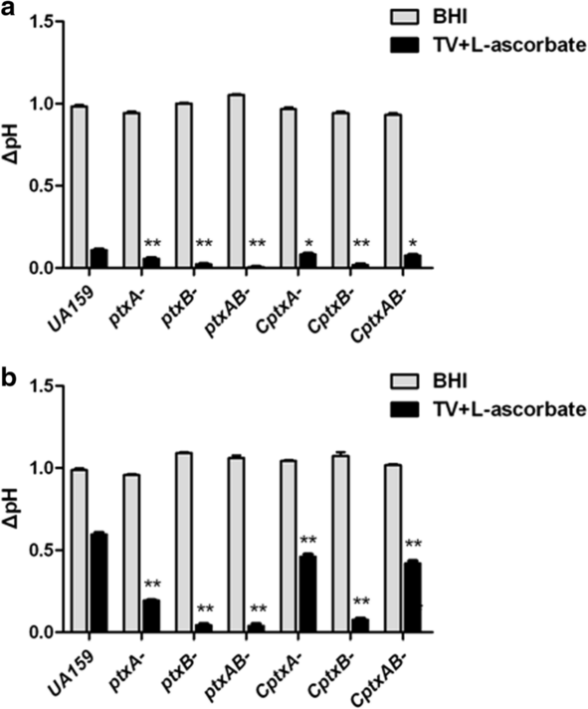
Acid production assay. Wild-type UA159, *ptxA* ^*−*^ strain, *ptxB* ^*−*^ strain, *ptxAB* ^*−*^ strain, *CptxA* ^*−*^ strain, *CptxB* ^*−*^ strain and *CptxAB* ^*−*^ strain were incubated in BHI or TVL medium for 24 h (**a**) and 48 h (**b**) anaerobically. The pH measurements of the media were performed before and after the incubation, and are presented as ΔpH. A significant difference is indicated by *P < 0.05, **P < 0.01 compared to UA159. The results presented here are the average of three independent experiments performed in triplicate

As seen in the results of the acid production assay (Fig. 2), the wild-type UA159 and all mutant derivatives grew well and acidified the medium to about the same terminal pH in BHI medium after both 24 h and 48 h of incubation. The ΔpH of the BHI medium was almost 0.95. However, in the case of the TVL medium, the terminal pH slightly decreased for all strains after incubation. The three mutants, and especially the *ptxB*^*−*^ and *ptxAB*^*−*^ strains, lowered the pH to a level significantly lower than that observed in wild-type UA159 culture after 24 h of incubation (*P <* 0.01). The ΔpH of the TVL medium that *ptxA*^*−*^ strain, *ptxB*^*−*^ strain and *ptxAB*^*−*^ strain grown in were 0.0567 ± 0.0115, 0.0233 ± 0.0100 and 0.0067 ± 0.0057, respectively. Furthermore, complemented strains recovered their acid production capacity, with the exception of the *CptxB*^*−*^ strain. The reduced growth of the *CptxB*^*−*^ strain may account for its negligible pH change. Results that after 48 h of incubation were the same, except that all strains had produced more acid and the ΔpH of the medium was greater than it was at 24 h.

### *ptxA*^*−*^ and *ptxB*^*−*^ mutants did not survive an acid shock

To determine the effects of *ptxA* and *ptxB* deletions on the ability to tolerate acid stress, the wild-type UA159 and single or double mutants were incubated in TVL medium with a pH of 5.0 for 1 h to induce an adaptive acid tolerance response and then were subjected to acid killing with a low-pH buffer (pH 2.8). However, none of the mutant strains formed colonies on the assay plates following 15 min of low-pH incubation in triplicate tests, showing that the acid shock caused serious damage to the mutants.

### Inactivation of the *ptxA* and *ptxB* genes affects biofilm and EPS formation in TVL medium

**Fig. 3:**
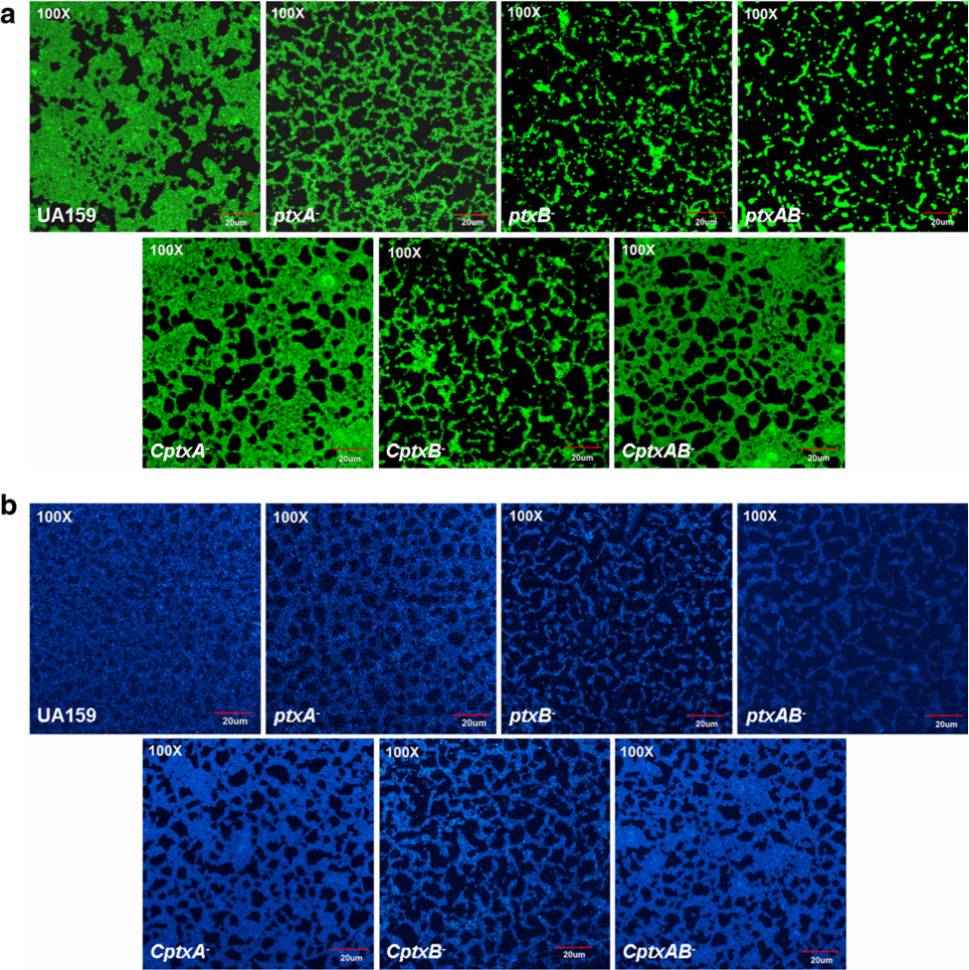
Biofilm and EPS formation analysis with confocal laser scanning fluorescence microscopy. Wild-type UA159, *ptxA* ^*−*^ strain, *ptxB* ^*−*^ strain, *ptxAB* ^*−*^ strain, *CptxA* ^*−*^ strain, *CptxB* ^*−*^ strain and *CptxAB* ^*−*^ strain were incubated in TVL medium for 120 h anaerobically. The formed biofilms were stained with SYTO9 (**a**) and EPS were stained with calcofluor white (**b**). Confocal laser scanning fluorescence microscopy was used to examine. At least five independent fields were collected at 100× magnification per experiment and three independent experiments were performed. Red lines represent 20 μm

It could be seen from the results of confocal laser scanning fluorescence microscopy analysis, biofilms stained with the fluorescent dye SYTO9 appeared green (Fig. 3a) and EPS stained with calcofluor white appeared blue (Fig. 3b). The cover area of the biofilms formed by *S. mutans* UA159 and its derivatives was shown in Table 2. When using L-ascorbate as the sole carbon source, wild-type UA159 formed both small and large amorphous microcolonies and covered 65.93 % of the surface. It created a thick and complex biofilms structure with a large amount of EPS. However, the biofilms and EPS *ptxA*^*−*^ strain formed were sparser and much thinner than UA159. It covered only 39.61 % of the surface, but it still could form network structure. *ptxB*^*−*^ and *ptxAB*^*−*^ strains formed much less prolific biofilms with only small amounts of EPS, in which cells were scattered on the surface as chains and the biofilms were too thin to form three-dimensional structure.

**Table 2:** Cover area of the biofilms formed by *S. mutans* UA159 and its derivatives

	UA159	*ptxA* ^*−*^	*ptxB* ^*−*^	*ptxAB* ^*−*^	*CptxA* ^*−*^	*CptxB* ^*−*^	*CptxAB* ^*−*^
Cover area (%)	65.93	39.61	24.24	18.57	62.16	31.97	56.01

They covered only 24.24 and 18.57 % of the surface respectively. Complementation in strains *CptxA*^*−*^ and *CptxAB*^*−*^ restored biofilms and EPS formation to a level similar to that of wild-type UA159. However, *CptxB*^*−*^ strain could not restore the wild-type phenotype.

### Transcriptional analysis of *ptxA*, *ptxB* and their operon

**Fig. 4:**
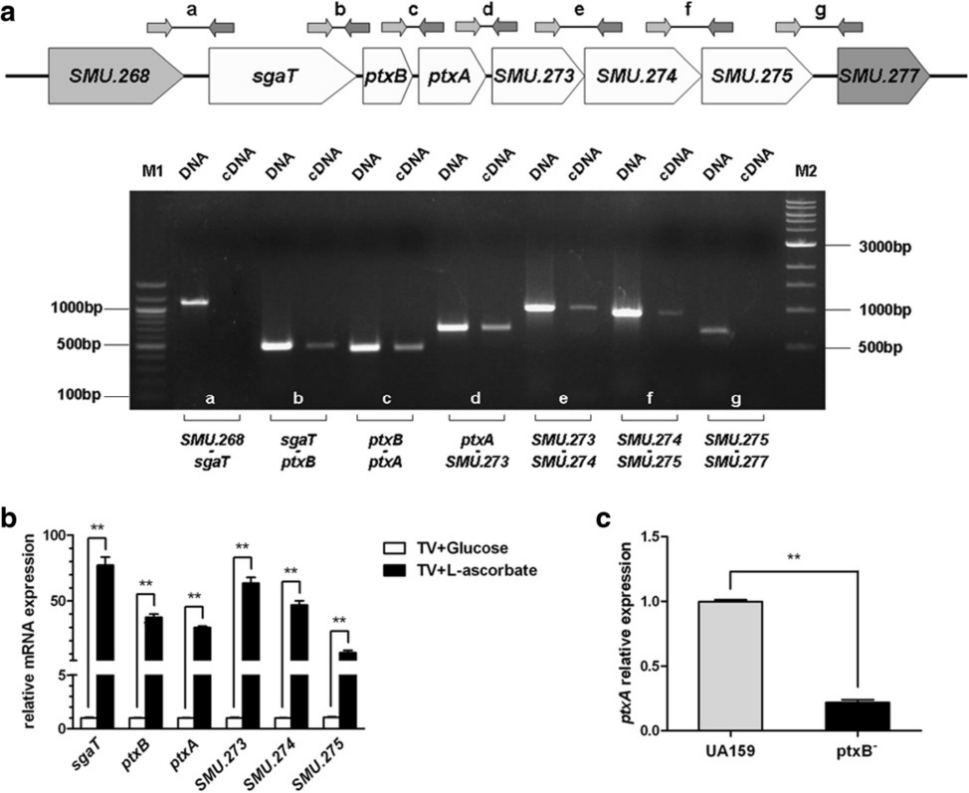
Transcription evaluation of *ptxA*, *ptxB* and their operon. **a** PCR analysis of *ptxA*, *ptxB* and adjacent genes with specific primers that span the sequences *SMU*.268 to *sgaT*, *sgaT* to *ptxB*, *ptxB* to *ptxA*, *ptxA* to *SMU*.273, *SMU*.273 to *SMU*.274, *SMU*.274 to *SMU*.275 and *SMU*.275 to *SMU*.277 by using cDNA. The letters a-g correspond to the amplified regions illustrated above the agarose gel. Lanes: M1, 100 bp DNA Ladder; DNA, chromosomal DNA of UA159; cDNA, cDNA of UA159; M2, 1 kb DNA Ladder. **b** Quantitative real-time PCR (qRT-PCR) analysis of the influence of L-ascorbate on the transcription levels of *ptxA*, *ptxB* and adjacent genes. The results are presented as relative mRNA expression. Significant differences are indicated by **P < 0.01. **c** Expression of *ptxA* gene in wild-type UA159 and *ptxB* ^*−*^ strain evaluated by qRT-PCR. There was a statistically significant difference between these two strains (**P < 0.01). The qRT-PCR results presented here are the average of three independent experiments performed in triplicate

Transcriptional analysis using cDNA templates and primers that spanned the adjacent genes showed amplified bands in b, c, d, e and f regions (Fig. 4a) indicating that *ptxA*, *ptxB,* and the upstream gene *sgaT*, the downstream genes *SMU*.273, *SMU*.274 and *SMU*.275 are parts of the same operon. However, *SMU*.268 and *SMU*.277 are not parts of it. Quantitative real-time PCR (Fig. 4b) demonstrated that, compared with the gene expression in cells grown in medium containing glucose, the transcription level of these genes in cells grown in the presence of 15 mM L-ascorbate were all elevated significantly (*P* < 0.01), further revealing that *ptxA*, *ptxB,* and the adjacent genes *sgaT, SMU*.273, *SMU*.274 and *SMU*.275 are parts of the same operon. In addition, the higher transcription levels of *ptxA* and *ptxB* genes in TV medium containing only L-ascorbate reinforced the finding that *S. mutans* could ferment L-ascorbate to obtain energy under anaerobic conditions, and suggested that the presence of L-ascorbate was required for up-regulation of transcription of *ptxA* and *ptxB*. However, once *ptxB* gene was knockout, the expression of *ptxA* gene decreased significantly (*P* < 0.01) compared with wild-type UA159 (Fig. 4c). This result could well explain the finding in bacterial growth rates that why the wild-type phenotype could not be restored in the *CptxB*^*−*^ strain.

## Discussion

Since the first discovery of PTS in *E. coli* [37], special efforts have been made to study the characteristics and functions of various PTS proteins in both gram-negative and gram-positive microorganisms, including *S. mutans*, the most common pathogen in dental caries. The presence of PTS involved in high-efficiency transport and phosphorylation of numerous carbohydrates largely accounts for the high cariogenicity of *S. mutans*. Apart from the two general proteins, EI and HPr, many genes coding for different carbohydrate-specific EII complexes of the PTS have been isolated and identified, such as the *scrA* gene for sucrose [38], the *mtlA* gene for mannitol [10], the *lacFE* genes for lactose [39], the *manLMN* genes for mannose [40], and others. In the present study, two genes, *ptxA* and *ptxB*, that were identified and presumed to be involved in anaerobic utilization of L-ascorbate, were analyzed.

Similar to *E. coli* and some other enteric bacteria, *S. mutans* could grow in defined medium supplemented with L-ascorbate as the sole energy. This provided evidence that *S. mutans* can obtain energy by fermenting this compound in an anaerobic atmosphere. However, at high concentrations, L-ascorbate failed to support the growth of *S. mutans*. This may be the result of an alteration of the internal redox state of the cells [23]. L-ascorbate can trigger the Fenton reaction in the presence of redox-active iron and oxygen, which yields ROS from hydrogen peroxide and leads to oxidative stress [41, 42]. Moreover, although L-ascorbate is an effective antioxidant, H_2_O_2_ will be released from its oxidation and can cause damage to cells [43]. The deletion of the *ptxA* and *ptxB* genes seriously affected the growth of *S. mutans* when using L-ascorbate as the sole carbon source, which indicated that the *ptxA* and *ptxB* gene products are indeed involved in the anaerobic dissimilation of L-ascorbate. However, the deletion of *ptxB* caused a more severe impact on cell growth than deletion of *ptxA*, and the wild-type phenotype could not be restored in the *CptxB*^*−*^ strain. This suggests that the *ptxB* gene, or its product, seems to be more important in this metabolic process. Previous study has found that the interaction of PtxA and PtxB proteins of *S. mutans* is weak [28], which increased the complexity of the phosphoryl transfer mechanism of L-ascorbate-specific PTS of *S. mutans*. Based on our experimental results, we have reasons to believe that *ptxB* plays a more essential role in the phosphoryl transfer. Another reasonable interpretation is that deletion of the *ptxB* gene caused a polar effect on the downstream *ptxA* gene, in which case complementation by *ptxB* could not restore the phenotype.

*S. mutans* has the ability to produce organic acids and cause enamel demineralization, so acidogenic capacity plays a crucial role in the occurrence of caries. The acid production assay indicated that an absence of *ptxA* and *ptxB* genes leads to lower glycolytic activities. This weakened capacity for acidogenesis is likely attributed to the reduced ability to grow, as reflected by the reduced growth rates and culture densities. Additionally, the pH of the medium can also affect the ability of glycolytic activity to lower the external pH. At lower pH, the cellular metabolism and energy levels are lower due to repression of amino acid synthesis genes. In addition, both glycolytic activity and amino acid biosynthesis require NAD^+^ as a cofactor [44, 45]. Since the total intracellular NAD^+^ pool is limited, competition between these two processes for NAD^+^ should slow down both pathways [46].

It is well known that *S. mutans* possesses an acid-tolerance response and the ability to tolerate acid stress will elevate after initial incubation in low pH medium [47, 48]. However, according to the results from the acid killing assay, none of the three mutants could survive in a buffer with a pH of 2.8, even after a 1 h acid adaptation. This showed that absence of the *ptxA* and *ptxB* genes resulted in the loss of the ability to tolerate acid stress when using L-ascorbate as the sole carbon source. The possible reason may be that L-ascorbate is not the most optimistic carbon source for *S. mutans* to ferment. The strains, on one hand, were under nutritional stress, and on the other hand, were under acidic stress. Oral streptococci often encounter acid stress conditions in the oral cavity. Therefore, the ability to survive acidic conditions may play a crucial role in the growth of these bacteria. Proton extrusion by the membrane-associated F-ATPase is the primary mechanism employed by *S. mutans* to maintain intracellular pH homeostasis [49], and mutations in some genes can have an impact on the conformation of, and functional capacity to extrude protons by, the F-ATPase enzyme [33]. However, the mechanism by which the absence of the *ptxA* and *ptxB* genes hinders the acid tolerance response remains to be discovered.

Biofilm formation is an important pathogenic trait that allows bacteria to attach to and colonize the tooth surface. Glucosyltransferases (GTFs) and glucan-binding proteins (GBPs) always play important roles in the process. However, when using L-ascorbate as the sole carbon source, UA159 still could form prolific biofilms and EPS, with no sucrose or glucose exist. We speculated that the limited nutritional stress was responsible for the prolific biofilm and EPS formation [46]. In TVL medium, L-ascorbate was the only energy source to support growth, and this may have automatically triggered biofilm formation because *S. mutans* is adapted to biofilm formation as its primary life style. What’s more, the difference of biofilm and EPS formation capacity among wild-type strain, mutant strains and complemented strains is likely attributed to the difference of bacterial growth rates.

The carbohydrate-specific PTS catalyze the concomitant transport and phosphorylation of their sugar substrates [6]. So far, 45 homologous L-ascorbate phosphotransferase transport systems from a wide variety of bacteria have been identified [50]. These systems fall into five structural types, and in *S. mutans*, EIIA, EIIB, and EIIC are encoded by distinct genes. The *ptxA* and *ptxB* genes of *S. mutans* encode the putative EIIA and EIIB of the L-ascorbate-specific PTS, and *sgaT* encodes the putative EIIC. What’s more, the downstream gene *SMU*.273 encodes 3-keto-L-gulonate-6-phosphate decarboxylase, *SMU*.274 encodes L-xylulose 5-phosphate 3-epimerase, and *SMU*.275 encodes L-ribulose-5-phosphate 4-epimerase. They also play essential roles in L-ascorbate metabolism. Based on the PCR analysis using cDNA as template, *sgaT, ptxB*, *ptxA, SMU*.273, *SMU*.274 and *SMU*.275 appear be parts of the same operon. This is similar to the *ulaA-F* operon in *E. coli* [51] that encodes the three components of the L-ascorbate phosphotransferase transport system (*ulaABC*), as well as three catabolic enzymes (*ulaDEF*). The *ulaA*, *ulaB,* and *ulaC* gene products are involved in the uptake and phosphorylation of L-ascorbate, and the *ulaD*, *ulaE,* and *ulaF* gene products are involved in the subsequent metabolism by the pentose phosphate pathway [23, 24]. Based on the result that *sgaT, ptxA*, *ptxB, SMU*.273, *SMU*.274 and *SMU*.275 could be all up-regulated significantly in the presence of L-ascorbate, it was concluded in this study that L-ascorbate is a potential inducer of the operon.

## Conclusion

This work indicates that *ptxA* and *ptxB* genes, encode putative enzyme IIA and enzyme IIB of the L-ascorbate-specific PTS in *S. mutans*, influence the physiology and virulence of *S. mutans*, including the growth rate, the capacity of aciduricity, acidogenesis, and formation of biofilm and EPS when using L-ascorbate as the sole carbon source. In addition, the expression of *ptxA* is regulated by *ptxB. ptxA*, *ptxB,* and the adjacent genes *sgaT, SMU*.273, *SMU*.274 and *SMU*.275 are parts of the same operon, and L-ascorbate is a potential inducer of the operon. Functional analysis of genes in PTS of the primary cariogenic etiological agent is crucial to the prevention and treatment of dental caries. Current efforts are being directed toward gaining a better understanding of how these genes are regulated, and to reveal further insights into their roles in metabolic pathways.

### Availability of data and materials

The data supporting the conclusions of this article are included within the article and additional file.

## Additional file

Additional file 1: Table S1. Primers used in this study. (DOC 62 kb)

## Abbreviations

ABC transporters: ATP-binding cassette transporters
BHI: brain-heart infusion
CSP: competence-stimulating peptide
EI: enzyme I
EII: enzyme II
EPS: extracellular polysaccharides
GBPs: glucan-binding proteins
GTFs: glucosyltransferases
HPr: histidine-containing phosphocarrier protein
OD: optical density
PCR: polymerase chain reaction
PEP: phosphoenolpyruvate
PTS: phosphotransferase system
qRT-PCR: quantitative real-time PCR
Spe^r^: spectinomycin resistance
TV: tryptone-vitamin
TVG: tryptone-vitamin supplemented with glucose
TVL: tryptone-vitamin supplemented with L-ascorbate
