# Supplementary material for: Identification and functional analysis of the L-ascorbate-specific enzyme II complex of the phosphotransferase system in Streptococcus mutans
Source: BMC Microbiol. 2016 Mar 22;16:51. doi: 10.1186/s12866-016-0668-9 (PMC4802650; doi:10.1186/s12866-016-0668-9)
Supplement: Additional file 1: Table S1. — Primers used in this study. (DOC 62 kb) [file 12866_2016_668_MOESM1_ESM.doc]

**Table S1** Primers used in this study.

| **Name** | **Nucleotide sequence(5′-3′)** | **Application** |
| --- | --- | --- |
| ptxA-UP-F | CGGGATCCTATGATATTGTTGTCGCTTC | Upstream fragment for *ptxA^−^* strain |
| ptxA-UP-R | CCCAAGCTTAAAGGTAACGGGCTCTGT | Upstream fragment for *ptxA^−^* strain |
| ptxA-DOWN-F | CCGGCTGCAGTGACAGAGCCCGTTACCT | Downstream fragment for *ptxA^−^* and *ptxAB^−^* strain |
| ptxA-DOWN-R | GGAATTCCATATGTTTACGGGCTGCTTTCAT | Downstream fragment for *ptxA^−^* and *ptxAB^−^* strain |
| ptxB-UP-F | CGGGATCCTAGGTCTGGCAGGATTTG | Upstream fragment for *ptxB^−^* and *ptxAB^−^* strain |
| ptxB-UP-R | CCCAAGCTTATCCGATACACCTAATTGACG | Upstream fragment for *ptxB^−^* and *ptxAB^−^* strain |
| ptxB-DOWN-F | CCGGCTGCAGGATATTGTTGTCGCTTC | Downstream fragment for *ptxB^−^* strain |
| ptxB-DOWN-R | GGAATTCCATATGGTAACGGGCTCTGTCAATGT | Downstream fragment for *ptxB^−^* strain |
| ptxB-F | TAAGAATTCTTGTTTTATTATTAGAAAGGTGTTACAATTA | *ptxA^−^ ,ptxB^−^* and *ptxAB^−^* complementation |
| ptxA-R | AGGTCGACTCTAGAGGATCCTCATGAATCAAGGTCAAGCCCCTCA | *ptxA^−^* and *ptxAB^−^* complementation |
| ptxB-R | AGGTCGACTCTAGAGGATCCTTATTTCAATGCTTCTTCCAATTTTG | *ptxB^−^* complementation |
| SMU.268-sgaT-F | TGATAAAGGGAGACGG | PCR analysis, span SMU.268 and *sgaT* |
| SMU.268-sgaT-R | AAATGGGACGAAAGGT | PCR analysis, span SMU.268 and *sgaT* |
| sgaT-ptxB-F | GTTTATGCCGACAAA | PCR analysis, span *sgaT* and *ptxB* |
| sgaT-ptxB-R | CTTCGCCAACTGAAC | PCR analysis, span *sgaT* and *ptxB* |
| ptxB-ptxA-F | TATTGTTGTCGCTTCG | PCR analysis, span *ptxB* and *ptxA* |
| ptxB-ptxA-R | GTAACGGGCTCTGTC | PCR analysis, span *ptxB* and *ptxA* |
| ptxA-SMU.273-F | TTGACAGAGCCCGTTAC | PCR analysis, span *ptxA* and SMU.273 |
| ptxA-SMU.273-R | CGCTTGTGAAATACCG | PCR analysis, span *ptxA* and SMU.273 |
| SMU.273-274-F | ATGAAAGCAGCCCGTAA | PCR analysis, span SMU.273 and SMU.274 |
| SMU.273-274-R | GATTTGTGCCCAATGTAG | PCR analysis, span SMU.273 and SMU.274 |
| SMU.274-275-F | TCGCCGTTATCCACTG | PCR analysis, span SMU.274 and SMU.275 |
| SMU.274-275-R | AATACCGCCAACTTCC | PCR analysis, span SMU.274 and SMU.275 |
| SMU.275-277-F | CCGCAAGCACTCAAA | PCR analysis, span SMU.275 and SMU.277 |
| SMU.275-277-R | ATCAACTAAGGCACC | PCR analysis, span SMU.275 and SMU.277 |
| QsgaT-F | TCTAACAAACTTCTCCCTG | qPCR of *sgaT* |
| QsgaT-R | TGGAACGAAACCTGTG | qPCR of *sgaT* |
| QptxA-F | AGAGCCCGTTACCTTT | qPCR of *ptxA* |
| QptxA-R | CTTCTTCTGGCGTTTT | qPCR of *ptxA* |
| QptxB-F | CTTCGCCAACTGAAC | qPCR of *ptxB* |
| QptxB-R | CATGGGCTCATCAATG | qPCR of *ptxB* |
| QSMU.273-F | TTACAGCAGCGGTTTC | qPCR of SMU.273 |
| QSMU.273-R | CACGCACAGCATTATTT | qPCR of SMU.273 |
| Q SMU.274-F | CTATCGCAGCCCTTCA | qPCR of SMU.274 |
| Q SMU.274-R | GCGTTTCTTCTACCGTTT | qPCR of SMU.274 |
| Q SMU.275-F | ACGGAAGGCGTGTCT | qPCR of SMU.275 |
| Q SMU.275-R | TTTCTTTCGGCGTCA | qPCR of SMU.275 |
| Q16S rRNA-F | CACACCGCCCGTCACACC | qPCR of 16S rRNA |
| Q16S rRNA-R | CAGCCGCACCTTCCGATACG | qPCR of 16S rRNA |

Underlined nucleotides are restriction sites engineered for cloning
